# Machine learning-based models for predicting the efficacy and safety of recombinant human interleukin-11 in the treatment of cancer therapy-induced thrombocytopenia: exploration and preliminary validation from a multicenter retrospective study

**DOI:** 10.3389/fcell.2026.1882398

**Published:** 2026-07-09

**Authors:** Yingmei Wen, Jinxiong Xia, Yi Dong, Zheming Liu, Shuyang Yu, Yuanyuan Wang, Shuman Qing, Xinyi Li, Jingyi Miao, Dongling Tang, Yi Yao

**Affiliations:** 1 Cancer Center, Renmin Hospital of Wuhan University, Wuhan, China; 2 Department of Radiation Oncology, The Second Affiliated Hospital of Guangxi Medical University, Nanning, China; 3 Department of Clinical Laboratory, Renmin Hospital of Wuhan University, Wuhan, China; 4 Hubei Provincial Research Center for Precision Medicine of Cancer, Wuhan, China

**Keywords:** cancer therapy-induced thrombocytopenia (CTIT), machine learning, predictive model, random forest (RF), recombinant human interleukin-11 (rhIL-11)

## Abstract

**Introduction:**

Cancer therapy-induced thrombocytopenia (CTIT) is a common hematologic toxicity associated with anti-tumor treatment. Recombinant human interleukin-11 (rhIL-11), as a thrombopoietic agent, is widely used in clinical practice. However, its efficacy and safety exhibit substantial individual variability, and reliable tools for individualized prediction are currently unavailable.

**Methods:**

Based on real-world data from 12,431 CTIT patients treated with rhIL-11 across 58 Chinese centers (July 2023-June 2024), this study retrospectively collected comprehensive information on demographics, clinical characteristics, and treatment regimens. The Least Absolute Shrinkage and Selection Operator (LASSO) regression was applied to identify key clinical features. Six machine learning algorithms --logistic regression (LR), decision tree (DT), random forest (RF), support vector machine (SVM), extreme gradient boosting (XGBoost), and lightweight gradient boosting machine (LightGBM) --were used to construct separate models for predicting platelet response (efficacy) and adverse events (safety) following rhIL-11 treatment. The dataset was randomly divided into training and test sets in a 7:3 ratio, and an independent external validation set was used to assess model generalizability. SHapley Additive exPlanations (SHAP) analysis was used to provide visual explanations of model feature importance.

**Results:**

The RF model demonstrated superior performance in efficacy prediction, with an AUC of 0.812 (95% CI: 0.797-0.828) in the test set and 0.761 (95% CI: 0.717-0.805) in the external validation set; for safety prediction, the RF model also performed best, with an AUC of 0.796 (95% CI: 0.778-0.814) in the test set and 0.739 (95% CI: 0.695-83), in the external validation set. However, the relatively low specificity of RF models in predicting efficacy and safety limited their potential for practical clinical application. SHAP analysis revealed that the predominant factors influencing efficacy were chemotherapy, prior anti-tumor therapy, history of grade III myelosuppression, targeted therapy, and duration of rhIL-11 treatment; whereas key predictors of safety outcomes included immunotherapy, dosing frequency, chemotherapy, and CTIT grades.

**Discussion:**

This study validated that machine learning models (particularly the RF model combined with SHAP analysis) exhibit highly sensitive preliminary screening performance, while enhancing model interpretability. These findings may facilitate the effective identification of patients most likely to benefit from rhIL-11 and provide references for personalized, precision treatment decisions in CTIT management.

## 1 Introduction

Cancer therapy-induced thrombocytopenia (CTIT) is a common hematologic toxicity associated with anti-tumor treatment, which can lead to treatment delay, interruption, dose reduction, and even bleeding events, thereby compromising therapeutic efficacy and long-term survival (Kuter, 2022; Song et al., 2025). CTIT is defined as a peripheral blood platelet (PLT) count below 100 × 10^9^/L attributable to anti-tumor therapies (such as chemotherapy, radiotherapy, targeted therapy, and immunotherapy), with severity graded from I to IV according to the Common Terminology Criteria for Adverse Events (CTCAE) version 5.0 (Guideline Working Committee of Chinese Society of Clinical Oncology, 2025; National Cancer Institute, 2017). The incidence and severity of CTIT vary with tumor types, anti-tumor treatment regimens, comorbidities, and extent of bone marrow involvement (Mei et al., 2025). Current treatment strategies for CTIT primarily include PLT transfusion and thrombopoietic agents, the latter represented by recombinant human interleukin-11 (rhIL-11), recombinant human thrombopoietin (rh-TPO), and thrombopoietin receptor agonists (TPO-RAs) (Zhao et al., 2025). Although PLT transfusion is the most rapid, direct, and effective intervention for CTIT, its routine use is limited by blood product shortages, short duration of PLT count maintenance, and the risks of transfusion reactions and infections. Consequently, it is typically reserved for patients with PLT counts ≤10 × 10^9^/L (Humbrecht et al., 2018; Schiffer et al., 2018). RhIL-11 is one of the thrombopoietic agents approved by both the United States Food and Drug Administration (FDA) and the National Medical Products Administration (NMPA) of China for the treatment of CTIT. As a pleiotropic cytokine, it stimulates the proliferation and differentiation of hematopoietic stem cells and megakaryocyte precursors, while also promoting megakaryocyte maturation, thereby accelerating PLT production (Liu et al., 2020; Mei et al., 2025; Song et al., 2018). Although multiple studies, including our previous work, have confirmed the overall efficacy and safety of rhIL-11 in treating CTIT (Liu et al., 2020; Yang et al., 2025), considerable heterogeneity remains in its efficacy and safety across different real-world clinical settings. Therefore, developing personalized and precise treatment strategies for patients receiving rhIL-11 has become an urgent priority in the clinical management of CTIT.

In recent years, significant advances have been made in research on rhIL-11 for CTIT treatment. A cross-sectional study involving 1,437 Chinese patients with CTIT reported that 27% opted for monotherapy with rhIL-11 (Chen et al., 2024). Several clinical studies have further revealed the heterogeneity in the efficacy of rhIL-11, which is closely associated with tumor type, chemotherapy regimen, baseline PLT level, and individual patient characteristics (Yang et al., 2025; Zhang et al., 2022). However, effective tools for predicting treatment response to rhIL-11 remain unavailable, and clinical decision-making still relies primarily on empirical judgment, lacking sufficient precision. Therefore, identifying the key factors that influence the efficacy and safety of rhIL-11 and developing personalized predictive models have become major priorities in current research.

Machine learning has been increasingly applied in the medical field and has demonstrated notable advantages in the screening, diagnosis, and prognosis assessment of various diseases, particularly in predictive research related to malignant tumors (Swanson et al., 2023). The technology can extract and integrate clinically relevant information from large-scale datasets to support clinical decision-making (Capobianco, 2022). With the help of machine learning, it is expected that personalized and precise therapeutic decisions will become more achievable. However, despite their high predictive capabilities, machine learning models are often limited by their inherent “black box” nature, which makes it difficult to elucidate the specific contributions of each variable to the predictive results, thereby undermining their credibility and acceptability in clinical practice (Belle and Papantonis, 2021). The SHapley Additive Explanations (SHAP) method combines optimal credit allocation with local interpretability, intuitively illustrating the importance of each variable and thus generating more interpretable model outputs (Lundberg et al., 2020).

Therefore, this study aims to develop a machine learning-based predictive model using real-world data to identify CTIT patients likely to benefit from rhIL-11 treatment in terms of efficacy and safety, thereby providing decision support for personalized and precise clinical management. Additionally, SHAP analysis will be applied to enhance model interpretability and facilitate the identification of high-risk patients, thereby promoting early intervention in clinical practice.

## 2 Materials and methods

### 2.1 Study population

We retrospectively analyzed clinical data from 12,431 patients with malignant tumors who received rhIL-11 treatment for CTIT across 58 medical centers in China between July 2023 and June 2024. The data encompassed baseline demographic characteristics, medical history, tumor types, anti-tumor treatment regimens, rhIL-11 administration details, efficacy evaluations, and adverse events (AEs). This study was approved by the ethics committees of all participating centers and conducted in accordance with the principles of the Declaration of Helsinki (2013 revision). Given the retrospective design of the study, all data were de-identified prior to analysis, and the requirement for informed consent was waived by the ethics committees.

Inclusion Criteria: (1) Diagnosis of malignant tumor confirmed by pathology or cytology; (2) Receipt of anti-tumor treatments such as chemotherapy, radiotherapy, targeted therapy, or immunotherapy; (3) Development of thrombocytopenia (PLT count ≤100 × 10^9^/L) following treatment and subsequent treatment with rhIL-11; (4) Regular monitoring of PLT counts during treatment.

Exclusion Criteria: (1) Thrombocytopenia caused by non-anti-tumor treatment factors (e.g., chronic liver disease or infection); (2) Use of other PLT-elevating drugs; (3) Poor adherence to rhIL-11 treatment or discontinuous use; (4) Incomplete records or missing critical information.

### 2.2 Data extraction and preprocessing

We extracted relevant clinical characteristics from the data, including gender, age, weight, history of comorbidities (hypertension, coronary heart disease, diabetes, infectious diseases, history of surgery or trauma, and history of COVID-19 infection), history of prior myelosuppression (grades I to IV), tumor types (hematologic malignancy, lung cancer, gastric cancer, colorectal cancer, hepatic carcinoma, esophageal cancer, uterine malignancy, prostate cancer, breast cancer, pancreatic cancer, thyroid cancer, head and neck malignancy, urothelial cancer, and others), bone marrow involvement, history of prior anti-tumor therapy, history of bleeding, current chemotherapy, radiotherapy, targeted therapy, immunotherapy, surgery, chemotherapy regimens (platinum monotherapy, taxane monotherapy, anthracycline monotherapy, fluorouracil monotherapy, gemcitabine-based combination, platinum-based combination, anthracycline-based combination, taxane-based combination, and others), radiotherapy regimens (radiotherapy alone, concurrent chemoradiotherapy, and sequential chemoradiotherapy), baseline CTIT grades (grades I to IV), total days of rhIL-11 prescription, and dosing frequency (first-time, multiple sessions). The Least Absolute Shrinkage and Selection Operator (LASSO) regression was employed to identify key features while discarding irrelevant data. Multicategorical variables were included in the analysis after dummy variable transformation.

### 2.3 Treatment regimen

RhIL-11 was administered subcutaneously at a dose of 25–50 μg/kg/day until the PLT count reached ≥100 × 10^9^/L or increased by ≥ 50 × 10^9^/L from the baseline level.

### 2.4 Outcome evaluation metrics

The primary endpoint of this study is defined as the response rate, measured as the incidence of PLT count returning to 100 × 10^9^/L within 1 month of the first administration of rhIL-11; the secondary endpoint is the incidence of treatment-related AEs. AEs were classified and assessed according to the CTCAE v5.0, and mainly included fatigue, edema, gastrointestinal reactions, fever, rash, and atrial fibrillation, with severity graded from I to V.

### 2.5 Construction, validation, and evaluation of machine learning models

We randomly selected a medical center’s data as the external validation set, while the data from the remaining centers were randomly divided into training and testing sets in a 7:3 ratio (all data from each center was allocated to one set without internal splitting). A total of six machine learning models were constructed in this study: Extreme Gradient Boosting (XGBoost), Logistic Regression (LR), Random Forest (RF), Support Vector Machine (SVM), Lightweight Gradient Boosting Machine (LightGBM), and Decision Tree (DT). Five-fold cross-validation was used to perform hyperparameter tuning for the six models. The test set was not used during the model tuning phase but was reserved for model evaluation only after training was completed. The key hyperparameters for each model are detailed in Supplementary Tables S1 and S2.

To assess model performance, we compared various evaluation metrics, including the area under the ROC curve (AUC), accuracy, specificity, precision, recall, and F1-score, with the AUC serving as the primary evaluation metric to identify the machine learning model with the best predictive performance. Calibration curves were used to assess the consistency between actual and predicted outcomes, while decision curve analysis (DCA) was used to evaluate the net clinical benefit of each model.

### 2.6 Interpretability analysis

SHAP analysis was performed on the optimal predictive model to assess its interpretability. Based on Shapley values derived from game theory, SHAP calculates the contribution of each feature to the prediction results (Li et al., 2019). The importance of global features is ranked and presented in bar charts, highlighting the average influence of each feature on the model’s output. Additionally, waterfall plots and force plots were employed to interpret individual samples, revealing the direction and intensity of each feature’s influence on the prediction results, which helps in understanding the model’s decision-making process.

### 2.7 Statistical analysis

Statistical analysis and data visualization were performed using SPSS v29, R v4.5.0, and Origin. Continuous variables were presented as means and standard deviations, with group comparisons conducted using the Wilcoxon test or the Kruskal–Wallis test. Categorical variables were presented as frequencies and percentages, and group comparisons were performed using the χ^2^ test or Fisher’s exact test. The following R packages were used in this study for data preparation, analysis, and visualization, including: “dplyr” (v1.1.4), “data.table” (v1.17.4), “caret” (v7.0–1), “glmnet” (v4.1–9), “readxl” (v1.4.5), “openxlsx” (v4.2.8), “Matrix” (v1.7–3), “xgboost” (v1.7.11.1), “e1071” (v1.7–16), “randomForest” (v4.7–1.2), “lightgbm” (v4.6.0), “tree” (v1.0–44), “rpart” (v4.1.24), “partykit” (v1.2–24), “pROC” (v1.18.5), “ggplot2” (v3.5.2), “ggpubr” (v0.6.0), “rms” (v8.0–0), “dcurves” (v0.5.0), “ggbeeswarm” (v0.7.2), “shapviz” (v0.9.7), “fastshap” (v0.1.1). All statistical tests were two-tailed, and a P-value of <0.05 was considered statistically significant.

## 3 Results

### 3.1 Baseline information and clinical characteristics

Based on the inclusion and exclusion criteria, 12,431 CTIT patients treated with rhIL-11 were included in the analysis cohort. Among them, 7,653 were allocated to the training set, 3,274 to the test set, and 1,504 to the external validation set. Table 1 presents the baseline characteristics of the patients, including demographic details, medical history, tumor types, anti-tumor treatment regimens, and medication status. In this study, there were 1,463 patients with grade IV CTIT, distributed as follows: 666 in the training set, 632 in the test set, and 165 in the external validation set. Among all participants, 8,177 received chemotherapy, including 5,439 in the training set, 1,524 in the test set, and 1,214 in the external validation set.

**TABLE 1:** Baseline characteristics of patients.

Variables	Categories	Training set (n = 7653)	Test set (n = 3274)	External validation set (n = 1504)	*P*-value
Gender (%)	male	4316 (56.4)	1804 (55.1)	847 (56.3)	0.446
	female	3337 (43.6)	1470 (44.9)	657 (43.7)	
Age (%)	<60Y	3823 (50.0)	1993 (60.9)	914 (60.8)	<0.001
	≥60Y	3830 (50.0)	1281 (39.1)	590 (39.2)	
Weight	Median	62 kg	61 kg	60 kg	​
	mean ± SD	62.05 ± 9.73	61.51 ± 8.59	60.00 ± 8.02	<0.001
Medical history (%)	no	6791 (88.7)	2857 (87.3)	1454 (96.7)	<0.001
	yes	862 (11.3)	417 (12.7)	50 (3.3)	
Prior myelosuppression (%)	no	6238 (81.5)	2297 (70.2)	1227 (81.6)	<0.001
	Grade I	149 (1.9)	38 (1.2)	21 (1.4)	
	Grade II	555 (7.3)	240 (7.3)	83 (5.5)	
	Grade III	447 (5.8)	339 (10.4)	64 (4.3)	
	Grade IV	264 (3.4)	360 (11.0)	109 (7.2)	
Tumor type (%)	Hematologic malignancy	1958 (25.6)	1235 (37.7)	492 (32.7)	<0.001
	Solid tumor	5695 (74.4)	2039 (62.3)	1012 (67.3)	
	Lung cancer	1308 (17.1)	421 (12.9)	142 (9.4)	
	Gastric cancer	810 (10.6)	335 (10.2)	158 (10.5)	
	Colorectal cancer	955 (12.5)	278 (8.5)	176 (11.7)	
	Hepatic carcinoma	658 (8.6)	200 (6.1)	211 (14.0)	
	Esophageal cancer	275 (3.6)	165 (5.0)	26 (1.7)	
	Uterine malignancy	351 (4.6)	144 (4.4)	61 (4.1)	
	Prostate cancer	60 (0.8)	10 (0.3)	7 (0.5)	
	Breast cancer	408 (5.3)	219 (6.7)	37 (2.5)	
	Pancreatic cancer	171 (2.2)	34 (1.0)	7 (0.5)	
	Thyroid cancer	38 (0.5)	27 (0.8)	1 (0.1)	
	Head and neck malignancy	145 (1.9)	69 (2.1)	118 (7.8)	
	Urothelial cancer	57 (0.7)	14 (0.4)	9 (0.6)	
	Other	459 (6.0)	123 (3.8)	59 (3.9)	
Bone marrow involvement (%)	no	6694 (87.5)	3016 (92.1)	1290 (85.8)	<0.001
	yes	959 (12.5)	258 (7.9)	214 (14.2)	
Prior anti-tumor therapy (%)	no	6795 (70.4)	1922 (58.7)	926 (61.6)	<0.001
	yes	2858 (29.6)	1352 (41.3)	578 (38.4)	
Current surgery (%)	no	7350 (96.0)	3137 (95.8)	1425 (94.7)	0.072
	yes	303 (4.0)	137 (4.2)	79 (5.3)	
Current chemotherapy (%)	no	2304 (30.1)	1750 (53.5)	290 (19.3)	<0.001
	yes	5349 (69.9)	1524 (46.5)	1214 (80.7)	
Current radiotherapy (%)	no	6564 (85.8)	2865 (87.5)	1465 (97.4)	<0.001
	yes	1089 (14.2)	409 (12.5)	39 (2.6)	
Current targeted therapy (%)	no	6781 (88.6)	2583 (78.9)	1348 (89.6)	<0.001
	yes	872 (11.4)	691 (21.1)	156 (10.4)	
Current immunotherapy (%)	no	6700 (87.5)	3007 (91.8)	1398 (93.0)	<0.001
	yes	953 (12.5)	267 (8.2)	106 (7.0)	
Baseline CTIT grade (%)	Grade I	584 (7.6)	176 (5.4)	162 (10.8)	<0.001
	Grade II	2718 (35.5)	830 (25.4)	560 (37.2)	
	Grade III	3685 (48.2)	1636 (50.0)	617 (41.0)	
	Grade IV	666 (8.7)	632 (19.3)	165 (11.0)	
Dosing frequency (%)	First-time	5005 (65.4)	2161 (66.0)	985 (65.5)	0.828
	Multiple sessions	2648 (34.6)	1113 (34.0)	519 (34.5)	
Concurrent myelostimulatory drugs* (%)	no	6645 (86.8)	2837 (86.7)	1320 (87.8)	0.549
	yes	1008 (13.2)	437 (13.3)	184 (12.2)	
Bleeding history (%)	no	7402 (96.7)	3210 (98.0)	1375 (91.4)	<0.001
	yes	251 (3.3)	64 (2.0)	129 (8.6)	

* Myelostimulatory medicines include recombinant human granulocyte colony-stimulating factor and erythropoietin.

### 3.2 Construction and evaluation of efficacy prediction models

#### 3.2.1 Feature selection

**FIGURE 1:**
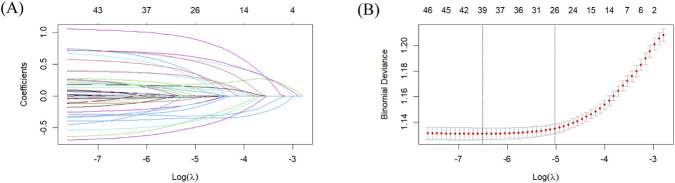
LASSO binary logistic regression was employed to analyze demographic information and clinical characteristics, and to identify key predictors of treatment efficacy. **(A)** Coefficient paths. **(B)** Cross-validation plot. When the regularization parameter is set to one standard deviation, the model has fewer features while maintaining its predictive performance.

The LASSO regression method was employed to identify key features, with iterative analysis conducted through 10-fold cross-validation. By selecting the lambda value corresponding to one standard error, 14 features closely associated with the therapeutic efficacy of rhIL-11 were identified from the training set: age, history of comorbidities, history of prior myelosuppression, tumor types, bone marrow involvement, history of prior anti-tumor therapy, history of bleeding, current chemotherapy, radiotherapy, targeted therapy, chemotherapy regimens, radiotherapy regimens, baseline CTIT grades, and total days of rhIL-11 prescription (Figure 1).

#### 3.2.2 Evaluation and comparison of model performance

**FIGURE 2:**
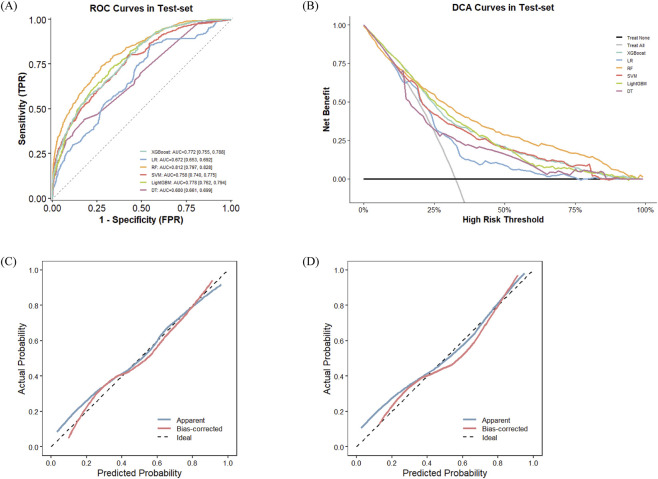
Comparative evaluation of six machine learning models for efficacy prediction. **(A)** ROC curves in the test set. **(B)** DCA curves in the test set. **(C)** Calibration curves of the RF model in the training set. **(D)** Calibration curves of the RF model in the test set.

Based on 14 key features, six machine learning models were constructed to predict the clinical efficacy of rhIL-11 treatment in patients with CTIT. After tuning the hyperparameters, each model was trained using the entire training set and subsequently evaluated in the test set. The discriminatory performance of each model in the test set is shown in Figure 2A. Among these models, the RF model exhibited the best performance, with an AUC of 0.812 (95% CI: 0.797–0.828). The remaining models also demonstrated good predictive ability, ranked in descending order of performance as follows: LightGBM (AUC = 0.778, 95% CI: 0.762–0.794), XGBoost (AUC = 0.772, 95% CI: 0.755–0.788), SVM (AUC = 0.758, 95% CI: 0.740–0.775), DT (AUC = 0.680, 95% CI: 0.661–0.699), and LR (AUC = 0.672, 95% CI: 0.653–0.692). To comprehensively evaluate model performance, we calculated accuracy, specificity, precision, recall, and F1-score (Supplementary Table S3). Comparative analysis demonstrated that the RF model outperformed other models across most metrics, with the highest accuracy (0.771), precision (0.784), recall (0.914), and F1-score (0.844), but relatively low specificity (0.477) (Supplementary Figure S1).

In terms of clinical applicability, decision curve analysis showed that all models demonstrated robust net benefit across most threshold probability ranges, with the RF model exhibiting the highest net benefit (Figure 2B). Furthermore, the calibration curve for the RF model demonstrated a good fit, indicating a high degree of consistency between the model’s predicted probabilities and actual outcomes, which further confirmed the reliability of its predictions (Figures 2C,D).

#### 3.2.3 Model performance in external validation set

**FIGURE 3:**
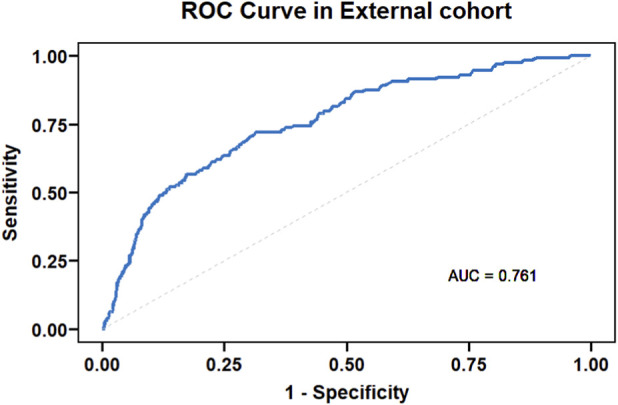
ROC Curve of the RF model for efficacy prediction in the external validation set.

The RF model also demonstrated favorable predictive performance for efficacy outcomes in the external validation set, with an AUC of 0.761 (95% CI: 0.717–0.805) (Figure 3).

### 3.3 Construction and evaluation of safety prediction models

#### 3.3.1 Feature selection

**FIGURE 4:**
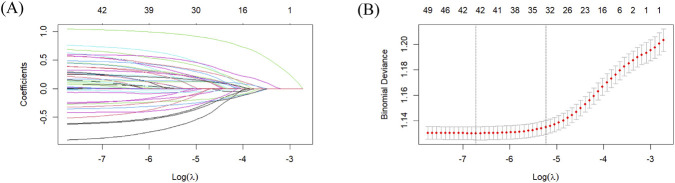
LASSO binary logistic regression was employed to analyze demographic information and clinical characteristics, and to identify key predictors of safety. **(A)** Coefficient paths. **(B)** Cross-validation plot. When the regularization parameter is set to one standard deviation, the model has fewer features while maintaining its predictive performance.

We used the LASSO regression method to identify key features and performed iterative analysis using 10-fold cross-validation. By selecting the lambda value corresponding to one standard error, we identified 14 features in the training set closely related to the safety of rhIL-11 treatment: age, weight, history of comorbidities, history of prior myelosuppression, tumor types, bone marrow involvement, history of prior anti-tumor therapy, current surgery, chemotherapy, targeted therapy, immunotherapy, chemotherapy regimens, dosing frequency, and baseline CTIT grades (Figure 4).

#### 3.3.2 Evaluation and comparison of model performance

**FIGURE 5:**
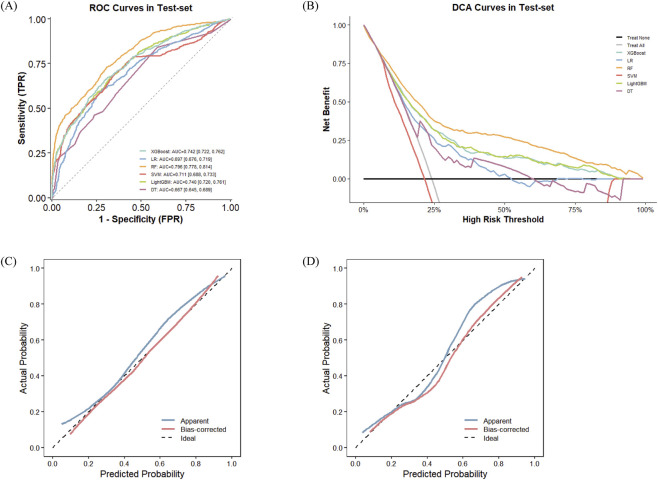
Comparative evaluation of six machine learning models for safety prediction. **(A)** ROC curves in the test set. **(B)** DCA curves in the test set. **(C)** Calibration curves of the RF model in the training set. **(D)** Calibration curves of the RF model in the test set.

Leveraging the 14 selected features, we constructed six machine learning models to predict the occurrence of AEs in CTIT patients treated with rhIL-11. The discriminatory performance of each model in the test set is shown in Figure 5A, with the RF model exhibiting superior performance and achieving an AUC of 0.796 (95% CI: 0.778–0.814). The remaining models also demonstrated certain predictive capabilities, ranked in descending order of performance as follows: XGBoost (AUC = 0.742, 95% CI: 0.722–0.762), LightGBM (AUC = 0.740, 95% CI: 0.720–0.761), SVM (AUC = 0.711, 95% CI: 0.688–0.733), LR (AUC = 0.697, 95% CI: 0.676–0.719), and DT (AUC = 0.667, 95% CI: 0.645–0.689). To comprehensively evaluate model performance, we calculated accuracy, specificity, precision, recall, and F1-score (Supplementary Table S4). Overall, the RF model performed best among all evaluated models, with the highest accuracy (0.821), precision (0.826), recall (0.968), and F1-score (0.891), although its specificity was relatively low (0.357) (Supplementary Figure S2).

With regard to clinical utility, decision curve analysis showed that models such as RF, LightGBM, and XGBoost demonstrated robust net benefit across most threshold probability ranges, with the RF model exhibiting the highest net benefit (Figure 5B). In addition, the calibration curve for the RF model demonstrated a good fit, indicating strong consistency between the predicted probability of AEs and the actual incidence rate, providing important insights into the model’s predictive reliability (Figures 5C,D).

#### 3.3.3 Model performance in external validation set

**FIGURE 6:**
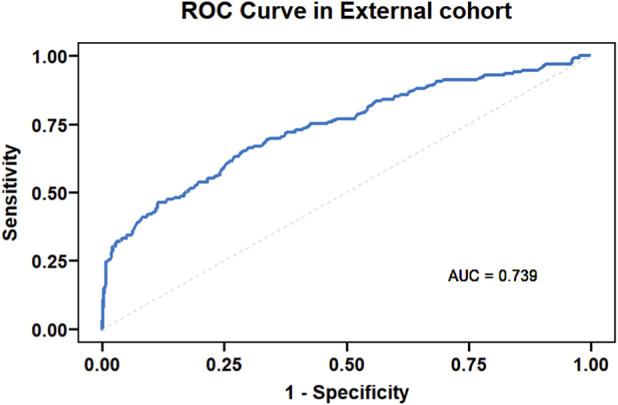
ROC Curve of the RF model for safety prediction in the external validation set.

In terms of safety prediction, the RF model continued to demonstrate good predictive performance in the external validation set, with an AUC of 0.739 (95% CI: 0.695–0.783), further confirming the model’s robustness (Figure 6).

### 3.4 Interpretability analysis of the efficacy and safety prediction models

#### 3.4.1 The efficacy prediction model

**FIGURE 7:**
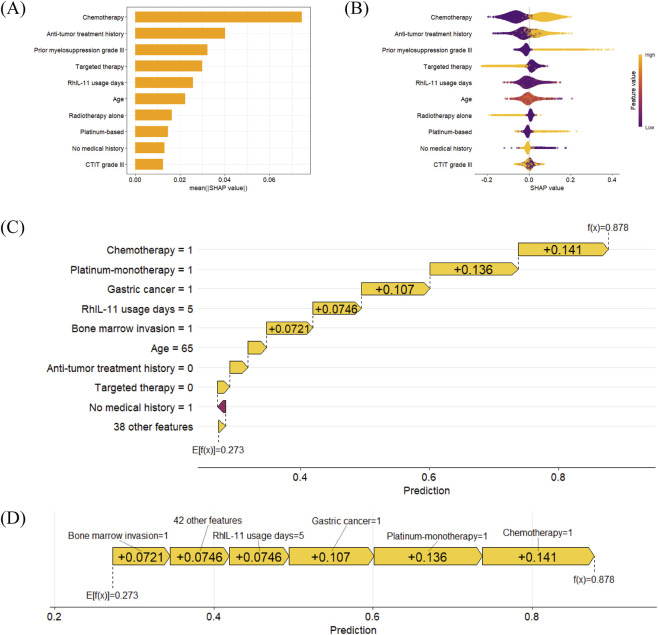
SHAP analysis of the RF model for efficacy prediction. **(A)** Ranking of the top 10 key features by importance score. **(B)** SHAP beeswarm plot: The impact of features on model outputs. **(C,D)** SHAP waterfall plot and single-sample force plot: Personalized prediction results for patients. Negative inhibitory variables and positive facilitative variables are represented by dark purple and yellow bars, respectively. The longer the bar, the more significant the functional significance.

To improve the interpretability of the RF model, SHAP analysis was performed to characterize the contribution of features to the prediction results at both the global and individual levels. In terms of treatment efficacy prediction, the higher the SHAP value of each feature, the greater its influence on PLT recovery following rhIL-11 treatment. As shown in Figure 7A, the global feature importance was ranked in descending order by median SHAP values of each variable in the RF model. The five features most strongly associated with efficacy prediction were: current chemotherapy, history of prior anti-tumor therapy, history of prior grade III myelosuppression, current targeted therapy, and total days of rhIL-11 prescription. The SHAP beeswarm plot further illustrated the direction and contribution of these features in the predictive model output, with yellow representing high feature values and purple representing low feature values (Figure 7B).

At the individual level, local explanation quantified and visualized the contribution of each feature to the prediction of a specific sample. This approach not only explained the nonlinear relationship between features and prediction outcomes but also provided traceable evidence for personalized prediction, thereby enhancing the credibility of model-based decision-making in clinical applications (Ding et al., 2026; Shu et al., 2024). Through a randomly selected patient as an example, the SHAP waterfall plot demonstrated that current chemotherapy (+0.141), a platinum-based chemotherapy regimen (+0.136), and gastric cancer (+0.107) were the major positive factors to PLT recovery in this patient (Figure 7C). In this plot, yellow indicates a positive contribution, purple indicates a negative contribution, and the length of each bar represents the magnitude of the contribution. E [f(x)] represents the SHAP baseline value, namely, the average model prediction, whereas f(x) refers to the SHAP output for the individual sample, corresponding to the actual predicted probability for that patient (Guan et al., 2024). By accumulating SHAP values, the plot provides an intuitive view of how the final prediction is generated, aiding in a deeper understanding of the decision-making logic of the machine learning models. Additionally, the single-sample force plot (Figure 7D) further illustrates the cumulative formation process underlying the efficacy prediction for this patient.

#### 3.4.2 The safety prediction model

**FIGURE 8:**
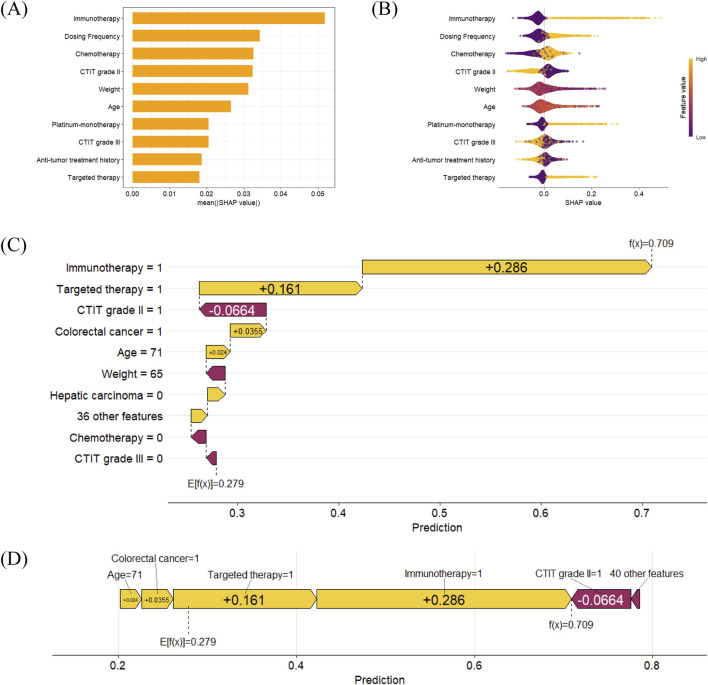
SHAP analysis Results of the RF model for safety prediction. **(A)** Ranking of the top 10 key features by importance score. **(B)** SHAP beeswarm plot: The impact of features on model outputs. **(C,D)** SHAP waterfall plot and single-sample force plot: Personalized prediction results for patients. Negative inhibitory variables and positive facilitative variables are represented by dark purple and yellow bars, respectively. The longer the bar, the more significant the functional significance.

In terms of safety prediction, the higher the SHAP value of a feature, the greater its influence on the occurrence of adverse reactions to rhIL-11 treatment. The global importance of features in the RF model is shown in Figure 8A, where the features’ contribution to the model is sorted in descending order by median SHAP value. The top five features most strongly associated with safety prediction were: current immunotherapy, dosing frequency, chemotherapy, grade II CTIT, and weight. The SHAP beeswarm plot visualizes the direction and extent of these features in the predictive model, with yellow representing high feature values and purple representing low feature values (Figure 8B).

At the individual level, by randomly selecting a patient sample for local interpretation, the SHAP waterfall plot illustrated the contribution of each feature to the prediction of adverse reactions for that patient. Among these, factors such as current immunotherapy (+0.286) and targeted therapy (+0.161) were the primary positive predictors of adverse reactions, while grade II CTIT and weight exhibited negative contributions (Figure 8C). Furthermore, the single-sample force plot (Figure 8D) provided a more intuitive visualization of the cumulative formation process of the safety prediction results for this patient.

## 4 Discussion

RhIL-11 is one of the key hematopoietic agents widely used in the treatment of CTIT and has been in clinical practice for over 20 years (Yang et al., 2025; Zhang et al., 2022). However, heterogeneity in real-world efficacy and the risk of adverse reactions remain critical challenges, complicating clinical decision-making and limiting the personalized application of this agent (Gong et al., 2024; Liu et al., 2019). Previous studies have predominantly focused on comparisons between rhIL-11 and other hematopoietic agents or pharmacoeconomic evaluations. Nevertheless, there is a lack of predictive tools to guide individualized treatment strategies that can adequately balance therapeutic benefits and risks (Shoukier et al., 2021; Zeylabi et al., 2023). Based on real-world data from 12,431 patients across 58 medical centers, we developed and validated predictive models for both the clinical efficacy and safety of rhIL-11 in CTIT management. This work not only confirmed the clinical effectiveness of rhIL-11 in the large-scale population but also identified key factors influencing treatment outcomes by employing interpretable machine learning methods, providing crucial guidance for personalized clinical treatment decisions.

Among the models evaluated for predicting therapeutic efficacy, the RF model demonstrated superior performance on the test set (AUC = 0.812), consistently outperforming other models in terms of accuracy, precision, recall, and F1-score. Furthermore, it also maintained good predictive performance on the external validation set (AUC = 0.761), demonstrating its generalizability. SHAP analysis further identified the main features influencing the efficacy of rhIL-11, including current chemotherapy, history of prior anti-tumor therapy, previous grade III myelosuppression, current targeted therapy, and total days of rhIL-11 prescription. Chemotherapy primarily induces chemotherapy-induced thrombocytopenia (CIT) by damaging and suppressing hematopoietic stem cells and megakaryocytes (Gao et al., 2023; Kuter, 2022). RhIL-11 acts on the aforementioned target cells to promote PLT production (Zhao et al., 2025). Notably, our findings indicated that patients with a history of prior anti-tumor therapy responded better to rhIL-11. This suggests that the bone marrow microenvironment shaped by previous treatment may be associated with responsiveness to subsequent hematopoietic agents. The underlying mechanism may be that repeated anti-tumor treatments select for stem cells with stronger anti-apoptotic capacity and relatively preserved hematopoietic reserve, which are more readily driven to proliferate or mature by hematopoietic agents during PLT depletion. Previous studies have indicated that a history of prior myelosuppression is a significant risk factor for the development of CTIT (Zhao et al., 2025), consistent with our findings and further quantifying the impact of this factor on the efficacy of rhIL-11. Of note, total days of rhIL-11 prescription, as a modifiable factor, provides a potential entry point for optimizing clinical dosing regimens and maximizing therapeutic outcomes.

For safety prediction, the RF model also achieved the best performance, with an AUC of 0.796 in the test set, and its accuracy, precision, recall, and F1-score reached 0.821, 0.826, 0.968, and 0.891, respectively. SHAP analysis revealed that current immunotherapy, dosing frequency, current chemotherapy, CTIT grades, weight and age are the key features for predicting AEs. In particular, the association between immunotherapy and the risk of AEs warrants special attention. In recent years, with the widespread adoption of immunotherapy, immune checkpoint inhibitor-induced thrombocytopenia (ICI-IT) has attracted increasing attention in clinical practice. ICI-IT is commonly observed in melanoma and non-small cell lung cancer. Although relatively uncommon, ICI-IT can be life-threatening, and its pathogenesis involves T-cell-mediated immune responses and autoantibody production (Jotatsu et al., 2018; Kroll et al., 2022; Moore et al., 2024; Tang et al., 2021). Furthermore, in addition to its hematopoietic-promoting activity, rhIL-11 also exerts immunomodulatory effects involved in the regulation of inflammation and immune responses. Consequently, in the context of immunotherapy, the safety management of rhIL-11 treatment must take into account more complex drug interactions to avoid an increased risk of AEs. Preclinical studies of rhIL-11 have shown that repeated administration results in cumulative toxic effects, yet the overall safety profile remains manageable (Yu et al., 2018), which is consistent with the results of this study. Recent chemotherapy exposure often causes multi-organ damage involving the heart, kidneys, liver, and bone marrow (Kuderer et al., 2022; Li et al., 2026; Lustberg et al., 2023; Zeien et al., 2022), leading to reduced functional reserve and compensatory capacity. This subsequently decreases the body’s tolerance to rhIL-11 and increases the risk of adverse reactions. Given that the current chemotherapy is associated with both efficacy and an increased risk of AEs, the benefit-risk ratio must be carefully weighed when using rhIL-11 to treat CTIT. The dual influence of age and weight further highlights the importance of individualized dosing, suggesting that adjusting the dose based on body surface area may be an effective strategy for optimizing the risk-benefit ratio.

In terms of the modeling strategy, this study established efficacy as the primary endpoint and safety as the secondary endpoint, constructing separate predictive models for each. This design enhances the specificity of the features for each endpoint, reduces the risk of overfitting associated with joint modeling, and better aligns with the needs of multidimensional clinical assessment. Although this strategy cannot automatically capture the intrinsic relationships between endpoints, combining it with SHAP analysis can elucidate these interconnections, thereby providing a basis for clinically precise treatment benefit-risk balancing.

Notably, although the RF model performs well overall in predicting both efficacy and safety, its specificity remains relatively low. Based on this, the RF model tends to yield a high false-positive rate, limiting its accuracy in directly guiding personalized treatment. The relatively low specificity may be attributed to several factors, including insufficient feature discriminative power, suboptimal threshold selection, and inherent limitations of the study design and dataset. Negative samples may exhibit high clinical heterogeneity, with a more dispersed feature distribution, making it difficult for the model to learn a consistent classification pattern. In addition, the default probability threshold (0.5) currently used in the model may not be the optimal choice; adjusting this threshold appropriately is likely to achieve a better balance between recall and specificity. Furthermore, given the retrospective nature of the data, inherent biases and missing variables may have constrained the model’s ability to capture detailed nuances of real-world clinical decision-making. Future research could further enhance the model’s overall discriminatory ability through optimizing feature selection, adjusting the probability threshold, and conducting multi-center validation studies, thereby better meeting practical clinical needs.

Furthermore, while selecting an external validation set on a per-center basis approximates real-world cross-center heterogeneity, it also results in significant differences among the training, testing, and external validation sets across multiple key clinical characteristics, thereby introducing systematic selection bias. This baseline discrepancy may lead to an overestimation or underestimation of model performance in external validation. The external validation performance metrics should be interpreted as conservative estimates, reflecting both generalization error and distributional shift, and should not be directly equated with expected real-world performance in unobserved clinical settings. Therefore, the interpretation of external validation results requires careful consideration of the potential impact of baseline differences on performance estimates. Nevertheless, the model still maintains above-average discriminative performance on the external validation set despite these imbalances. It is recommended that future studies incorporate center effects as covariates into the modeling algorithm or optimizing data partitioning strategies to obtain more reliable assessments of generalizability.

This study has several strengths. Firstly, the large sample size and multi-center data substantially enhance the generalizability and extrapolation of the results, while the diversity of the study population enables the model to capture broader clinical characteristics. Secondly, the application of multiple machine learning algorithms overcomes the limitations of the linear assumptions inherent in traditional statistical models, better reflecting the complex non-linear relationships found in clinical data. Moreover, the integration of SHAP explainability analysis quantifies the magnitude and direction of the effects of key features on the predictive results, offering clinicians an intuitive basis for result interpretation and identification of potential intervention targets.

However, some limitations should be acknowledged. The retrospective design may introduce selection bias. Certain clinical variables (such as PLT counts prior to the next treatment cycle and laboratory parameters) were not included in the analysis, which may result in the omission of some key variables. In addition, the documentation of AEs relies on real-world case data, which may be subject to potential under-reporting or incomplete documentation. In the future, further large-scale, prospective studies should be conducted to validate the model’s accuracy and its generalizability to other populations. Clinical variables from a wider range of dimensions should be incorporated to continuously improve the model’s predictive accuracy, and the feasibility of integrating it into clinical decision support systems should be explored, such as developing web-based calculators or integrating it into hospital information systems to enable real-time treatment recommendations and risk assessment.

In summary, machine learning-based predictive models developed and validated in this study help identify patient subgroups likely to benefit from rhIL-11 treatment for CTIT, as well as potential safety risks. The RF model demonstrates reasonable predictive performance and generalizability, suggesting its potential as a preliminary screening tool with high clinical sensitivity. The key characteristics further identified by SHAP analysis (such as anti-tumor therapy regimens, CTIT grades, and history of prior myelosuppression) serve as an evidence-based foundation for developing stratified treatment strategies and identifying high-risk populations. With the integration of multi-omics data and the advancement of artificial intelligence technologies, we look forward to developing more precise predictive models to drive the transition of CTIT treatment towards precision medicine.

## Data Availability

The raw data supporting the conclusions of this article are not publicly available due to ethical and privacy restrictions related to clinical patient data, but are available from the corresponding authors upon reasonable request and with appropriate institutional approval.
