# Supplementary material for: Machine learning-based models for predicting the efficacy and safety of recombinant human interleukin-11 in the treatment of cancer therapy-induced thrombocytopenia: exploration and preliminary validation from a multicenter retrospective study
Source: Front Cell Dev Biol. 2026 Jul 9;14:1882398. doi: 10.3389/fcell.2026.1882398 (PMC13391855; doi:10.3389/fcell.2026.1882398)
Supplement: Supplementary file 3 [file Supplementaryfile1.doc]

**Supplementary Table 1: Key Hyperparameters of the Efficacy Prediction Models**

| **Algorithms** | **Hyperparameters** | **Settings** |
| --- | --- | --- |
| XGBoost | gamma max_depth  eta colsample_bytree  min_child_weight  subsample  nrounds | 0.1 6  0.1  0.8  3  0.8 100 |
| LR (glm) | C max_iter epsilon | NA 25 1e-8 |
| RF | mtry ntree nodesize | 10 500 1 |
| SVM | gamma cost | 0.05 1 |
| LighGBM | num_leaves max_depth  min_data_in_leaf  feature_fraction  learning_rate lambda_l1 lambda_l2 scale_pos_weight min_gain_to_split | 31 -1 20 0.8 0.1 0 0 1 0 |
| DT | cp max_depth  minsplit  minbucket xval | 0.0013 30 20  7 10 |

**Supplementary Table 2: Key Hyperparameters of the Safety Prediction Models**

| **Algorithms** | **Hyperparameters** | **Settings** |
| --- | --- | --- |
| XGBoost | gamma max_depth  eta colsample_bytree  min_child_weight  subsample  nrounds | 0.1 6  0.1  0.8  3  0.8 100 |
| LR (glm) | C max_iter epsilon | NA 25 1e-8 |
| RF | mtry ntree nodesize | 8 500 1 |
| SVM | gamma cost | 0.1 0.5 |
| LighGBM | num_leaves max_depth  min_data_in_leaf  feature_fraction  learning_rate lambda_l1 lambda_l2 scale_pos_weight min_gain_to_split | 31 -1 5 0.8 0.05 0 0 1 0 |
| DT | cp max_depth  minsplit  minbucket xval | 0.0022 30 20  7 10 |

Abbreviations: XGBoost, extreme gradient boosting; LR, logistic regression; RF, random forest; SVM, support vector machine; LightGBM, light gradient boosting machine; DT, decision tree

**Supplementary Table 3. Performance Metrics of Each Treatment Efficacy Prediction Model in The Test Set**

| **Model** | **AUC (95% Cl)** | **Accuracy** | **Specificity** | **Precision** | **Recall** | **F1 score** |
| --- | --- | --- | --- | --- | --- | --- |
| XGBoost | 0.772 (0.755, 0.788) | 0.744 | 0.931 | 0.712 | 0.356 | 0.474 |
| LR | 0.672 (0.653, 0.692) | 0.583 | 0.452 | 0.429 | 0.855 | 0.571 |
| RF | 0.812 (0.797, 0.828) | 0.771 | 0.477 | 0.784 | 0.914 | 0.844 |
| SVM | 0.758 (0.740, 0.775) | 0.740 | 0.364 | 0.751 | 0.912 | 0.827 |
| LightGBM | 0.778 (0.762, 0.794) | 0.739 | 0.407 | 0.759 | 0.898 | 0.823 |
| DT | 0.680 (0.661, 0.699) | 0.699 | 0.825 | 0.545 | 0.436 | 0.484 |

**Supplementary Table 4. Performance Metrics of Each Safety Prediction Model in The Test Set**

| **Model** | **AUC (95% Cl)** | **Accuracy** | **Specificity** | **Precision** | **Recall** | **F1 score** |
| --- | --- | --- | --- | --- | --- | --- |
| XGBoost | 0.742 (0.722, 0.762) | 0.791 | 0.947 | 0.643 | 0.302 | 0.411 |
| LR | 0.697 (0.676, 0.719) | 0.689 | 0.713 | 0.404 | 0.612 | 0.487 |
| RF | 0.796 (0.778, 0.814) | 0.821 | 0.357 | 0.826 | 0.968 | 0.891 |
| SVM | 0.711 (0.688, 0.733) | 0.789 | 0.304 | 0.810 | 0.942 | 0.871 |
| LightGBM | 0.740 (0.720, 0.761) | 0.803 | 0.959 | 0.705 | 0.312 | 0.432 |
| DT | 0.667 (0.645, 0.689) | 0.692 | 0.770 | 0.380 | 0.445 | 0.410 |

**Supplementary Figure 1. Performance Metrics of Each Treatment Efficacy Prediction Model in The Test Set**

**Supplementary Figure 2. Performance Metrics of Each Safety Prediction Model in The Test Set**
